# 

**Published:** 1921-06-11

**Authors:** 


June 11, 1921] [Price Twopence

### The Hospital

The Workers' Newspaper of Administrative Medicine and Institutional Life, Administration, National Insurance and Health.

CONTENTS

The Cave Report ... ... ... ... ... 173

Official Advice ... ... ... ... ... ... 173

The Voluntary Hospitals Committee ... ... 174

Notes of the Week ... ... ... ... ... 175

The Hospital Crisis—The Birthday Honours—Hospitals and Coal Supply—A Suggestion for Hospital Sunday—Vacant Hospital Secretaryships—The History of *X*-rays—Another Hint to the Metropolis—The Langley Memorial Prize—"Eating" Grass—A Presentation—The late Sir James Horlick, Bart.—Oil Cookers in Hospitals—Hospital Conditions in Vienna.

Alimentary Investigation ... ... ... ... 177

Some Essentials in Diagnosing Abdominal Cases.

The Widening Fields of Public Health ... ... 178

The History of Medicine ... ... ... ... 179

.he Spirit of Charity and its Origin.

Women's Work in the X-Ray Department ... 180

I.—Refinements in Radiography.

The Public Well-Being ... ... ... ... ... 181

A Blind Policy—Call in the Doctor—Recreation and Juvenile Crime—The Death-rate of Mothers.

The Editor's Letter-Box ... ... ... ... 183

The Dangerous Drugs Hospital Scheme—Mr. Keyser's Proposal.

Passing Events and Topics ... ... ... ... 183

The Cirencester Experiment ... ... ... ... 184

The Matrons' and Sisters' Department ... ... 185

The Forthcoming State Register.
A New International Nursing Course.

Round the Hospitals ... ... ... ... ... 186

Registration Rules in Print Shortly ... ... 188

Meeting of the General Nursing Council for England and Wales.

The College of Nursing (Limited by Guarantee) vi

Forthcoming Events ... ... ... ... ... vi

